# Intervention Effects of a School-Based Health Promotion Programme on Obesity Related Behavioural Outcomes

**DOI:** 10.1155/2014/476230

**Published:** 2014-09-01

**Authors:** Susanne Kobel, Tamara Wirt, Anja Schreiber, Dorothea Kesztyüs, Sarah Kettner, Nanette Erkelenz, Olivia Wartha, Jürgen M. Steinacker

**Affiliations:** Division of Sports and Rehabilitation, Department of Internal Medicine II, Ulm University Medical Centre, Frauensteige 6, Haus 58/33, 89075 Ulm, Germany

## Abstract

Studies have shown preventive effects of an active lifestyle during childhood on later life; therefore, health promotion has to start early. The programme “Join the Healthy Boat” promotes a healthy lifestyle in primary school children. In order to evaluate it, children's behaviours in respect of increased physical activity (PA), a decrease in screen media use (SMU), more regular breakfast, and a reduction of the consumption of soft drinks (SDC) were investigated. 1943 children (7.1 ± 0.6 years) participated in the cluster-randomised study and were assessed at baseline and 1736 of them at follow-up. Teachers delivered lessons, which included behavioural contracting and budgeting of SMU and SDC. Daily SMU, PA behaviours, SDC, and breakfast patterns were assessed via parental questionnaire. After one-year intervention, significant effects were found in the intervention group for SMU of girls, children without migration background, and children with parents having a low education level. In the control group, second grade children skipped breakfast significantly more often. Tendencies but no significant differences were found for PA and SDC. This intervention seems to affect groups, which are usually hard to reach, such as children of parents with low education levels, which shows that active parental involvement is vital for successful interventions.

## 1. Introduction

One of the rising concerns in Western countries is the high prevalence of childhood obesity which has mainly been attributed to a constant decrease in physical activity levels and increased energy intake [1, 2]. Although recent research suggests a stabilisation in prevalence rates of overweight and obese children in developed countries [3], evidence shows that once obesity is established, it is problematic to reverse [4]. Additionally, it has been shown that obesity during youth is likely to follow through to adulthood [5].

Correspondingly, childhood obesity has been pronounced the main childhood health issue in developed countries [6] with consequences for the physical as well as psychological well-being for the affected children. Hence, obesity during childhood is a risk factor for subsequent chronic diseases in later life which should not be neglected [7, 8].

Sufficient physical activity and a well-balanced diet on the other hand are essential for normal growth and development [9] and play an important role in the prevention of increased weight and obesity [10]. Research shows that children lead an active lifestyle because of factors which they acquired as habits in early life and therefore profit from health benefits in adulthood [11]. Also, skipping breakfast is associated with higher rates of overweight and obesity in children [12] and especially the consumption of sugar-sweetened beverages has been identified as the most consistent dietary factor, which is associated with subsequent increases in weight status and fatness in children [13].

Healthcare professionals, governments, and many communities have long recognised childhood obesity as an increasing health problem and therefore have developed various programmes targeting inappropriate weight gain by reducing energy-dense foods and sedentary time (mainly television viewing) as well as increasing the daily amount of physical activity children engage in [14, 15].

Since several studies have shown positive and preventive effects of an active lifestyle during childhood on later life [7, 16, 17] and also that sedentary behaviour in childhood is maintained as an adult [11, 18] health promotion has to start early in life.

Therefore, schools have been identified as providing an ideal environment for the promotion of health-enhancing behaviours [19]. Based on the results of a recent review, Waters et al. [20] suggest that for interventions to be successful, they have to be integrated into the school curriculum and include amongst others “healthy eating, physical activity, and body image” [20, page 128] as well as support for teachers and parents. Furthermore, interventions intended to last longer than one year are more likely to become integrated into curriculum, school and parents activities than shorter interventions [21] and therefore are more promising to increase knowledge and behaviours which contribute to a healthy lifestyle and enhanced quality of life in the long term.

One programme incorporating those aspects is “Join the Healthy Boat - Primary School.” This low-threshold programme promotes a healthy lifestyle in primary school children in Baden-Württemberg, southwest Germany, and started in 2009 (for more detailed information see [22]). The programme's contents and materials are integrated into the primary school curriculum focusing on health promoting behaviour change towards more physical activity, less time spent with screen media, and a more healthy diet, especially targeting a reduction of soft drink consumption and breakfast skipping. The teaching materials, developed in collaboration with experienced primary school teachers, are delivered by the classroom teacher and promote healthy and active alternatives, which children are offered to choose in order to lead a healthier lifestyle. The prepared, ready-to-use teaching units include lessons that increase awareness (e.g., about the amount of sugar in some drinks), teach health-related topics such as “why does my body need physical activity?” and offer ideas and alternatives for leisure activities children can engage in without the use of screen media.

In order to know whether the implementation and intended outcomes were achieved a large-scale evaluation had to be carried out. The purpose of this study, therefore, is to investigate the children's behaviours after a one-year intervention in respect of the programme's key aspects: an increase of physical activity, a decrease in time spent with screen media as well as more regular breakfast, and a reduction of the consumption of sugar-sweetened beverages.

## 2. Materials and Methods

### 2.1. Intervention and Evaluation Design

The evaluation of this school-based, teacher-centred intervention, which is based on Bandura's social cognitive theory [23], is a prospective, stratified, cluster-randomised, and longitudinal study including an intervention group and a control group. After baseline measurements had been taken, the programme's intervention was carried out in the intervention group whereas the control group followed the regular school curriculum. Follow-up measurements were taken after one year.

The intervention is based on teaching materials offering action alternatives for recreational activities (without screen media), physical activity, and a healthy diet (focussing on breakfast and soft drinks) which are integrated into the primary school curriculum. The contents are delivered by the classroom teacher after taking part in a tripartite training course. Further detailed information on teaching materials and their contents have been published elsewhere [22]. In order to recruit the participating school and pupils, all primary schools of the state of Baden-Württemberg (southwest Germany) received written information about the programme and the intervention study, asking teachers to participate. Interested teachers then contacted the study group. Participation in the programme was voluntary and participating teachers had to agree with the randomisation process.

Stratification of randomisation was carried out on grade level based on information about the distribution of participating teachers within the different schools. Stratification according to number of classes and grade levels was realised on six different levels. Cluster-randomisation was carried out on school level into intervention and control groups. A detailed insight of the randomisation and stratification is provided elsewhere [22].

Approval for the study was obtained from the University's Ethics Committee, the Ministry of Culture and Education, and was provided in accordance with the Declaration of Helsinki. In addition, the study is registered at the German Clinical Trials Register (DRKS-ID: DRKS00000494).

### 2.2. Participants

1943 primary school children (7.1 ± 0.6 years; 51.2% male) in 154 classes (80 classes in the intervention group; 74 classes in the control group), who participated in the evaluation study of the programme, were assessed at baseline (Autumn 2010) and 1736 of them at follow-up (Autumn 2011). Prior to data collection, parents provided written and informed consent and children provided their assent to take part in the study.

### 2.3. Instruments

Anthropometric measurements such as children's height (cm) and body mass (kg) were taken by trained technicians according to ISAK Standards [24] using a stadiometer and calibrated electronic scales (Seca 213 and Seca 862, resp., Seca Weighing and Measuring Systems, Hamburg, Germany). The children's BMI was calculated as weight divided by height squared and converted to BMI percentiles (BMIPCT) using German reference data [25] to define their weight status. Cut-off points for overweight children were determined above the 90th percentile and for obese children above the 97th percentile.

All other parameters such as daily screen media time, physical activity behaviours, soft drink consumption and breakfast patterns as well as parental education levels, height, and body weight were assessed using a parental questionnaire. The included questions were based on the German Health Interview and Examination Survey for Children and Adolescents (KiGGS), which recently assessed health behaviour in 18,000 German children and adolescents [26]. Parental weight status was classified using WHO standards [27] with a cut-off point of 25 kg/m^2^ defining overweight.

### 2.4. Data Analysis

Statistics were performed using SPSS Statistics 21 (SPSS Inc., Chicago, IL, USA) with a significance level set to *α* < 0.05. Descriptive statistics were calculated (mean values and standard deviations). For categorical data, Fisher's exact test was used for the detection of group differences at baseline. For inference statistical analysis, physical activity was dichotomised by engagement on most days per week (i.e., four days or more) of at least 60 minutes of moderate to vigorous physical activity (MVPA). Time using screen media (TV, PC, and game consoles) was dichotomised using a cut-off point of one hour per day based on the recommendations of the American Academy of Paediatrics [28]. Parental data providing information on soft drink consumption were dichotomised by consuming soft drinks more than once versus less than once per week (median split). The frequency of having breakfast prior to going to school was also dichotomised as “often/always” versus “never/rarely.” Subsequently, logistic regression adjusted for baseline measures was used to determine odds ratios (OR) for all health outcomes.

## 3. Results

A summary of the participant's baseline sociodemographic, anthropometric, and lifestyle characteristics is shown in Table 1. No significant gender differences were found for height, weight, and BMIPCT. The prevalence of overweight including obesity is 9.0% and of obesity alone 4.0% of children.

Group comparing to check if the randomisation was successful revealed no differences between control and intervention groups for all relevant variables with the exception of migration background, which was significantly higher in the intervention group (*P* ≤ 0.01).

### 3.1. Physical Activity

At baseline, children engaged in 60 minutes of MVPA on 2.74 (±1.66) days per week. Further, 31.9% and 22.2% of boys and girls, respectively, spent at least 4 days per week being moderately to vigorously physically active for at least 60 minutes. 4.2% of children reached the 60 minutes of MVPA on seven days per week, which are recommended by the WHO [29]. At baseline, no differences between control and intervention groups were observed. Boys, however, showed significantly more activity than girls (*P* = 0.001).

At follow-up, children engaged in 60 minutes of MVPA on 2.82 (±1.61) days per week and 34.1% and 21.5% of boys and girls, respectively, spent at least 4 days per week being moderately to vigorously physically active for at least 60 minutes. 3.7% of children reached the recommended 60 minutes of MVPA on seven days per week.

Also, after one year, no significant differences in the amount of physical activity were found between control and intervention groups (Table 3).

However, there is a tendency towards more physical activity in the intervention group and a slight reduction of physical activity in the control group (Table 2). This tendency was especially pronounced if only considering boys, although statistical significance was not reached (OR = 1.34, *P* = 0.083, 95% CI [0.96; 1.88]).

### 3.2. Screen Media Consumption

Baseline results of screen media use show that 15.4% and 11.2% of boys and girls, respectively, spent a minimum of one hour per day using screen media, including television, computer/laptop, and video games. Boys spent significantly more time with screen media than girls (*P* = 0.01). No group differences at baseline between control and intervention group could be observed.

After one year, the proportion of children using screen media for at least one hour daily remained virtually unchanged with 15.6% of boys and 11.5% of girls. The gender difference, which could be observed at baseline, persisted, but examining the entire cohort, the intervention showed no significant effects on the time children spend in front of screen media (Table 3).

Nevertheless, there is a tendency towards less screen media use in the intervention group, whereas the opposite trend could be observed in the control group (Table 2).

Further, considering girls and boys separately, there is a significant difference between control and intervention groups with only girls in the intervention group using significantly less screen media per day than their counterparts in the control group (OR = 0.58, *P* = 0.04, 95% CI [0.35; 0.96]). Additionally, significant positive intervention effects on screen media consumption have been found in children (boys and girls) without a migration background as well as in children whose parents have a low education level (OR = 0.61, *P* = 0.043, 95% CI [0.38; 0.98] and OR = 0.64, *P* = 0.032, 95% CI [0.43; 0.96], resp.).

### 3.3. Soft Drink Consumption and Breakfast

Investigating children's soft drink consumption, at baseline, 24.6% of boys and 22.6% of girls drank sugar-sweetened beverages at least once per week. Neither a significant gender difference nor a difference between control and intervention groups could be observed at baseline.

Similarly, at follow-up, there was no significant difference between control and intervention groups (Table 3). Even though, a reduction of soft drink consumption could be seen in both groups. However, the decline in the intervention group was by trend greater than that in the control group (Table 2).

Data on children's breakfast behaviour show that at baseline 12.9% of children went to school without or rarely having breakfast before they leave. There was a significant gender difference with 15.4% of girls and 10.6% of boys skipping breakfast prior to school (*P* = 0.001).

At baseline and at follow-up, no significant differences between control and intervention group were found. Nonetheless, a tendency towards more children skipping breakfast could be observed in the control group at follow-up whereas in the intervention group the number of children who went to school without breakfast remained virtually the same (Table 2).

However, considering children in grade one and grade two separately, this trend becomes a significant difference for children in grade two: the second-graders in the control group skipped breakfast significantly more often than those in the intervention group (OR = 0.52, *P* = 0.024, 95% CI [0.30; 0.92]).

## 4. Discussion

This cluster-randomised effectiveness trial of a low-threshold, teacher-centred health promotion intervention led to a significant decrease of screen media use in girls and children without migration background compared to children receiving no intervention. “Join the Healthy Boat” also significantly improved children's breakfast behaviours in second grade and led to a tendency towards more overall physical activity in the intervention group.

Apart from that tendency, no significant effects in children's regular physical activity could be observed after the first year of this school-based intervention, which is consistent with previous interventions [30–32]. However, since physical activity is a primary determinant of optimal growth and health in children [33] and school has been determined as an important environment for physical activity [19], numerous recent studies and interventions have tried to increase children's activity levels during the past years. The approaches and methods of those interventions—as well as their results—differ widely, including the placement of a full-time member of staff in the schools, who is dedicated to facilitating healthy living [33] or the use of a so-called buddy system where older peers deliver health messages [34].

The present programme aimed at children changing their activity behaviours because of the choices they make, without reminders or additional PE sessions. Although, previous research has shown that social environmental factors such as teacher encouragement are positively related to children's physical activity levels [35, 36]. The “Join the Healthy Boat” intervention, however, focuses on delivering alternatives, so children learn about different ways and activities to spend their free time more actively. A longer lasting and more intense intervention might have shown more positive physical activity results, which was suggested by Ploeg and colleagues [33], comparing an intervention lasting one year compared to three years.

Another target of this intervention was to reduce children's sedentary time using screen media. Significant positive intervention effects were found in girls and children without a migration background as well as in children whose parents have a low education level but not boys or children with migration background. This is in accordance with a recent meta-analysis of 16 intervention programmes trying to reduce children's screen time which showed that around 60% of interventions result in positive effects on children's sedentary time [37]. The authors also noted that—the same as in this study—all programmes combined the reduction of screen time with other components. It was highlighted that for a successful intervention and reduction of screen time parental involvement is vital [37] and current research suggests that interventions show better results if they include a family component [38]. Apart from offering children active alternatives for sedentary behaviour, in the “Join the Healthy Boat” programme, screen time reduction was mainly targeted by letters to parents and the so-called family homework, which asked parents to spend a “screen-free weekend” with their children. In the letters, parents were introduced to TV guidelines and age-appropriate time limits for screen media use but were also offered alternatives of what to do on such a “screen-free weekend.” This may be one of the reasons why children without migration background benefited from this intervention compared to children with migration background. Although the letters to parents were provided in Turkish and Russian as well as German, parents from other countries may have not received or understood the given information. Similarly, to parents with a low educational level the given information and guidelines may have been news so they then might have actually tried to control their children's screen media use to a certain extent. However, the intervention also showed significantly reduced screen media use in girls but not boys, which is contrary to other research [39]. But it has further been suggested that interventions as this one are effective in changing children's behavioural capability (which was not assessed in this study) but do not necessarily result in a shift in behaviour [39], which might explain the lack of overall effects regarding screen media use.

The third aspect of this programme was a reduction of sugar-sweetened beverages and breakfast skipping. In compliance with recent Danish research [40], no differences between the groups were observed in the amount of sugar-sweetened beverage intake, which is possibly due to the fact that soft drink consumption was only communicated to parents using letters and no family homework. Skipping breakfast, however, was tackled using family homework (having a healthy family breakfast together) as well as joint breakfast in class (twice a year). It is well known that parents play an important role in the development of healthy breakfast behaviours [41] and parental breakfast intake has been shown to be associated with the breakfast intake of their children [42]. Children in this study were having breakfast a little more regularly than that reported in other researches [12], where skipping breakfast was also associated with increased weight, which was not assessed in this study. In the present study, children in second grade skipped breakfast significantly less often than their counterparts in the control group showing positive intervention effects.

Since recent findings suggest that it is at or around the first school year when overweight in German children particularly increases [43], it is vital to start health promotion early. For school-based interventions the use of a comprehensive approach for health promotion is recommended [44] and Vasques et al. [45] suggest interventions that focus on children's physical activity as well as their diet and involve their parents in order to be successful.

Although this study has a large sample size, which increases the likelihood of having sufficient power to detect intervention effects, some aspects should be considered when interpreting these findings. The use of parental report measures of physical activity, screen media use, and drinking/eating behaviour and the associated recall biases is a limitation of this study. Furthermore, participating in this study may have led to an increased social desirability bias with regard to the measured variables as awareness was raised for the importance of physical activity and other health behaviours. Also, the present intervention was very low “dose” and delivered by regular class teachers rather than external staff which also may lower the likelihood of the “Hawthorne” or observer effect. Further it should be noted that the effects of health promotion are usually not detected in a short time frame such as the one of the present evaluation study. The “Join the Healthy Boat” intervention covers the entire period of primary school in Germany which lasts four years. In contrast, the corresponding study could only investigate one year because the waiting control group could not deny the intervention any longer. Even though a major strength of this study is the randomised controlled design with a control group, the teachers in that group were also very health conscious and have not been “inactive,” which led to a strong contamination with other efforts to promote pupils' health in the control group. Moreover, according to a microsimulation model, health gains from interventions targeting children occur in the long term [46].

## 5. Conclusions

Although, only using a low-dose teacher-centred approach, the school-based health promotion programme “Join the Healthy Boat” managed to achieve significant positive effects in the reduction of screen media use (in girls and children without migration background and parents with a low education level only) and breakfast skipping (second grade children only) as well as a tendency towards more physical activity in the intervention group. Whilst some effects were rather small, the intervention seems to affect even groups which are usually hard to reach such as children of parents with low education levels. This shows that active parental involvement is vital for a successful intervention and should be intensified and demanded.

Since most behaviours are difficult to change within one year, further research should include investigations into the level of intensity and length of time an intervention needs to be of to show lasting effects on behaviour change. Further, the kind and level of parental involvement would be of interest for future studies in order to improve health promotion programmes.

## Figures and Tables

**Table 1:** Baseline characteristics of participants in the “Join the Healthy Boat” study.

	Missing	Intervention	Control	Total
	Values	(*n* = 954)	(*n* = 782)	(*n* = 1736)
Age, years [m (sd)]		7.09 (0.63)	7.06 (0.63)	7.08 (0.63)
Boys, *n* (%)		475 (49.8)	411 (52.6)	886 (51.0)
Migration background, *n* (%)	244	280 (34.2)∗	183 (27.2)∗	463 (31.0)
Anthropometry				
BMI, [m (sd)]		16.03 (2.22)	15.92 (2.03)	15.98 (2.14)
BMIPCT, [m (sd)]		48.87 (27.82)	48.12 (27.49)	48.53 (27.67)
Overweight and obesity, *n* (%)		95 (10.0)	70 (9.0)	165 (9.0)
Parental characteristics				
Tertiary family educational level, *n* (%)	270	268 (33.2)	208 (31.6)	476 (32.5)
Overweight (mother), *n* (%)	301	247 (31.5)	195 (30.0)	442 (30.8)
Overweight (father), *n* (%)	393	460 (61.9)	355 (59.2)	815 (60.7)
Health and lifestyle characteristics				
MVPA on ≥4 days/week ≥60 min/day, *n* (%)	266	216 (26.8)	183 (27.6)	399 (27.1)
Screen media ≥1 h/day, *n* (%)	207	122 (14.5)	83 (12.0)	205 (13.4)
Soft drinks ≥1 time/week, *n* (%)	198	207 (24.5)	156 (22.5)	363 (23.6)
Skipping breakfast, *n* (%)	196	110 (13.0)	89 (12.8)	199 (12.9)

m (sd): mean (standard deviation); BMI: body mass index, BMIPCT: BMI percentiles, and MVPA: moderate to vigorous physical activity.

∗Significant difference, *P* ≤ 0.05.

**Table 2:** Baseline and follow-up results for physical activity, screen media consumption, soft drink consumption, and breakfast skipping.

	Intervention	Control	Total
	(*n* = 954)	(*n* = 782)	(*n* = 1736)
Physical activity^a^			
Baseline, *n* (%)	216 (26.8)	183 (27.6)	399 (27.1)
Follow-up, *n* (%)	231 (29.1)	177 (26.5)	408 (27.9)
Screen media consumption^b^			
Baseline, *n* (%)	122 (14.5)	83 (12.0)	205 (13.4)
Follow-up, *n* (%)	104 (12.7)	100 (14.6)	204 (13.6)
Follow-up (girls only)∗, *n* (%)	40 (9.8)	47 (14.2)	87 (11.3)
Follow-up (no migration background)∗, *n* (%)	49 (9.3)	62 (12.8)	111 (11.2)
Follow-up (low parental education)∗, *n* (%)	70 (13.9)	75 (17.3)	145 (16.1)
Soft drink consumption^c^			
Baseline, *n* (%)	207 (24.5)	156 (22.5)	363 (23.6)
Follow-up, *n* (%)	178 (21.8)	152 (22.1)	330 (22.0)
Breakfast skipping^d^			
Baseline, *n* (%)	110 (13.0)	89 (12.8)	199 (12.9)
Follow-up, *n* (%)	101 (12.4)	100 (14.5)	201 (13.4)
Follow-up (grade 2 only)∗, *n* (%)	42 (10.8)	53 (16.6)	95 (13.5)

^a^MVPA on ≥4 days/week ≥60 min/day (MVPA: moderate to vigorous physical activity); ^b^screen media ≥1 h/day; ^c^soft drinks ≥1 time/week; ^d^regular breakfast skipping.

∗Significant difference, *P* ≤ 0.05.

**Table 3:** Behavioural outcomes at follow-up for the intervention group.

	*n* ^a^	OR^b^	*P*	95% CI
Physical activity				
MVPA on ≥4 days/week ≥60 minutes MVPA	1386	1.18	0.19	[0.92, 1.52]
Screen media use				
Screen media ≥1 h/day	1471	0.75	0.10	[0.53, 1.06]
Soft drink consumption				
Soft drinks ≥1 time/week	1475	0.96	0.76	[0.72, 1.28]
Breakfast habits				
Skipping breakfast	1480	0.86	0.47	[0.58, 1.29]

OR: odds ratio, CI: confidence interval, and MVPA: moderate to vigorous physical activity; ^a^only cases with baseline and follow-up data; ^b^adjusted for baseline outcomes.
